# Integrative Advances for OCT-Guided Ophthalmic Surgery and Intraoperative OCT: Microscope Integration, Surgical Instrumentation, and Heads-Up Display Surgeon Feedback

**DOI:** 10.1371/journal.pone.0105224

**Published:** 2014-08-20

**Authors:** Justis P. Ehlers, Sunil K. Srivastava, Daniel Feiler, Amanda I. Noonan, Andrew M. Rollins, Yuankai K. Tao

**Affiliations:** 1 Ophthalmic Imaging Center, Cole Eye Institute, Cleveland Clinic, Cleveland, Ohio, United States of America; 2 Department of Biomedical Engineering, Case Western Reserve University, Cleveland, Ohio, United States of America; 3 Department of Biomedical Engineering, Lerner Research Institute, Cleveland Clinic, Cleveland, Ohio, United States of America; 4 Department of Ophthalmic Research, Cole Eye Institute, Cleveland Clinic, Cleveland, Ohio, United States of America; University of Florida, United States of America

## Abstract

**Purpose:**

To demonstrate key integrative advances in microscope-integrated intraoperative optical coherence tomography (*i*OCT) technology that will facilitate adoption and utilization during ophthalmic surgery.

**Methods:**

We developed a second-generation prototype microscope-integrated *i*OCT system that interfaces directly with a standard ophthalmic surgical microscope. Novel features for improved design and functionality included improved profile and ergonomics, as well as a tunable lens system for optimized image quality and heads-up display (HUD) system for surgeon feedback. Novel material testing was performed for potential suitability for OCT-compatible instrumentation based on light scattering and transmission characteristics. Prototype surgical instruments were developed based on material testing and tested using the microscope-integrated *i*OCT system. Several surgical maneuvers were performed and imaged, and surgical motion visualization was evaluated with a unique scanning and image processing protocol.

**Results:**

High-resolution images were successfully obtained with the microscope-integrated *i*OCT system with HUD feedback. Six semi-transparent materials were characterized to determine their attenuation coefficients and scatter density with an 830 nm OCT light source. Based on these optical properties, polycarbonate was selected as a material substrate for prototype instrument construction. A surgical pick, retinal forceps, and corneal needle were constructed with semi-transparent materials. Excellent visualization of both the underlying tissues and surgical instrument were achieved on OCT cross-section. Using model eyes, various surgical maneuvers were visualized, including membrane peeling, vessel manipulation, cannulation of the subretinal space, subretinal intraocular foreign body removal, and corneal penetration.

**Conclusions:**

Significant iterative improvements in integrative technology related to *i*OCT and ophthalmic surgery are demonstrated.

## Introduction

OCT has revolutionized the clinical practice of ophthalmology. OCT provides non-invasive high-resolution optical biopsy of tissue microstructure that facilitates disease diagnosis and guides therapeutic decision-making [1]. Seamless integration of this technology into ophthalmic surgery has the potential to be the foundation for a new paradigm in the surgical management of ophthalmic disease.

Over the last several years, intraoperative OCT (*i*OCT) has emerged as a viable adjunct to ophthalmic surgery. Anterior segment and vitreoretinal surgical procedures have been imaged with *i*OCT, and macular holes, vitreomacular traction, retinal detachment, and epiretinal membranes have all been visualized with *i*OCT in early foundational studies [2]–[9]. Intrasurgical feedback using *i*OCT during lamellar keratoplasty procedures has also been described [10]–[13].

Early applications of *i*OCT used portable OCT systems to provide intraoperative visualization either through a handheld, externally mounted, or microscope-mounted systems [2]–[4], [6]–[11]. Novel designs for handheld ophthalmic imaging systems have also been presented, which use state-of-the-art light-sources or multimodality imaging, and have potential applications in intraoperative imaging by improving imaging speed and ergonomics [14], [15]. Although these systems provide excellent visualization of the anatomic alterations that occur during surgery, they are limited by the “pause” required to bring the system into alignment, and “real-time” visualization of surgical maneuvers is not feasible with these systems.

In addition to microscope-mounted imaging systems, integrated approaches have also been presented that combine optics with prototype surgical instruments for intraoperative imaging and guidance. These include fiber probes [16], [17] for intraocular OCT imaging and integrated metrology and feedback systems for guiding instrument positions within the eye [18], [19]. Although these instruments may provide useful intraoperative feedback, their utility in guiding ophthalmic surgical maneuvers have not been demonstrated. Furthermore, fiber probe based instruments have traditionally been limited by inherent trade-offs in imaging field-of-view (FOV), sampling density, and resolution, and manual positioning and aiming of probes results in poor imaging stability, all of which needs to be addressed prior to clinical translation.

In order to address these limitations, microscope-integrated *i*OCT systems have been developed [5], [9], [20]–[23]. Preliminary integrated systems allowed for visualization of surgical instrumentation and instrument-tissue interactions [23]–[25]. However, the development of these landmark systems also revealed additional hurdles that must be overcome to achieve true seamless integration, including optimal ergonomic impact, heads-up display (HUD), improved focus control, and OCT-friendly surgical instrumentation [5], [20].

In this report, we describe several iterative advances in *i*OCT technology, including a second-generation microscope-integrated *i*OCT system, OCT-friendly surgical instrumentation, optimized dynamic *i*OCT imaging, and HUD visualization.

## Methods

### Microscope-Integrated iOCT System

**Figure 1:**
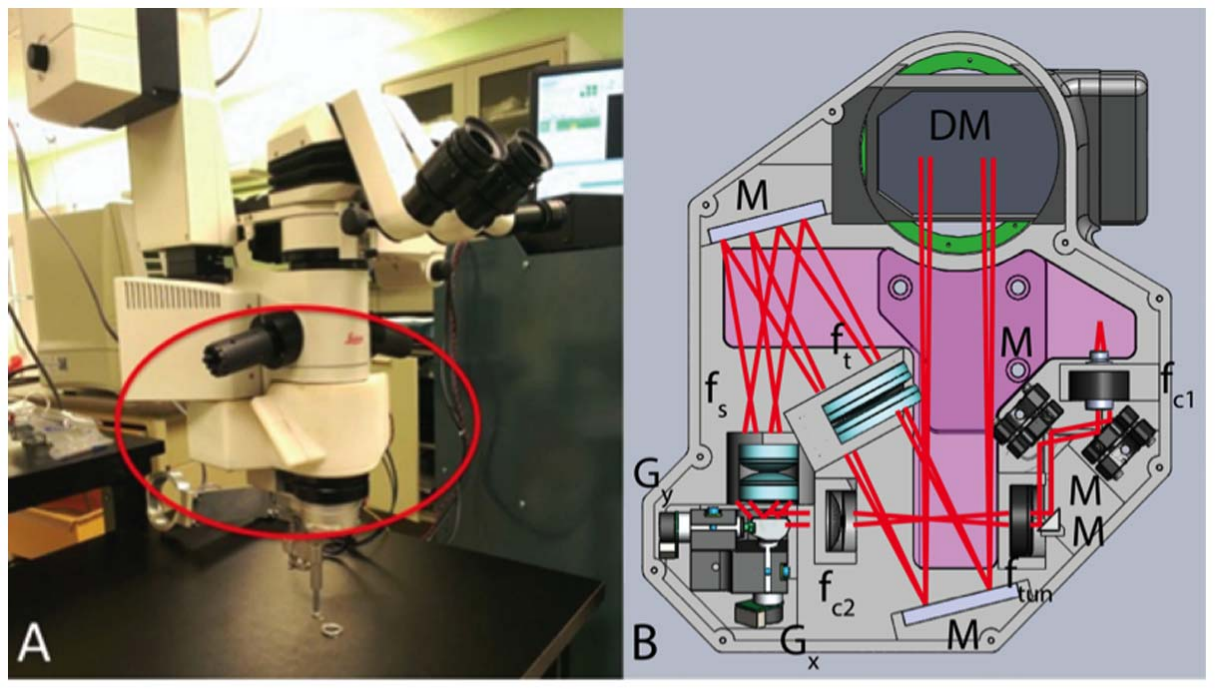
Microscope-integrated intraoperative OCT (*i*OCT) system. (A) Color photograph of system (red circle) on Leica 844 microscope. (B) Schematic of *i*OCT system, including tunable lens and monolithic design. DM, dichroic mirror; f, collimating, objective, scan, tube, and electrically tunable lenses; G, galvanometer scanners; M, fold mirrors.

The microscope-integrated *i*OCT system was implemented as an attachment to a Leica M844 (Wezlar, Germany) ophthalmic surgical microscope (Figure 1) [26]. The surgical microscope provides 3.5–21x motorized magnification for anterior segment viewing, and additional magnification for the posterior segment is set by the use of contact/non-contact ophthalmic lenses. An electrically tunable lens allowed for real-time focus control, which can compensate for ∼13.9 D of defocus for optimized imaging of both anterior and posterior segment and fine control of the OCT focus independent of the surgical view. Anterior segment *i*OCT visualization was achieved through the objective lens of the ophthalmic surgical microscope. Posterior segment *i*OCT visualization was obtained with either an Oculus BIOM3 (Wezlar, Germany) adapter for wide-field indirect ophthalmoscopy or contact lens for high magnification viewing (Volk, Mentor, OH). The relay optics in the custom-built scan-head focuses to a 24 µm spot during anterior segment imaging through a WD 200 mm objective lens or 1.4 mm collimated spot at the pupil for posterior segment imaging through a BIOM widefield ophthalmic lens (53565/WFE). All OCT images were acquired with 700 µW optical power at 830 nm center wavelength with 106 dB SNR at a line-rate of 50 kHz, a 5.2 µm axial resolution in air, and 1.14 mm 6 dB falloff. Pathlength changes for anterior and posterior segment imaging was adjusted by the imaging technician using a manually adjustable translation stage in the reference arm. Custom software (Bioptigen, Durham, NC) was utilized to perform real-time data acquisition, processing, archiving, and display.

Utilizing the DI C800 (Leica Microsystems, Heerbrugg, Switzerland), a VGA display was also folded into the optical path of the microscope ocular, which provided overlaid OCT images for a customizable HUD for surgeon feedback. Various approaches to HUD were tested for surgeon feedback, including aiming targets and live display of OCT cross-sectional data.

### OCT-Compatible Instrumentation

**Figure 2:**
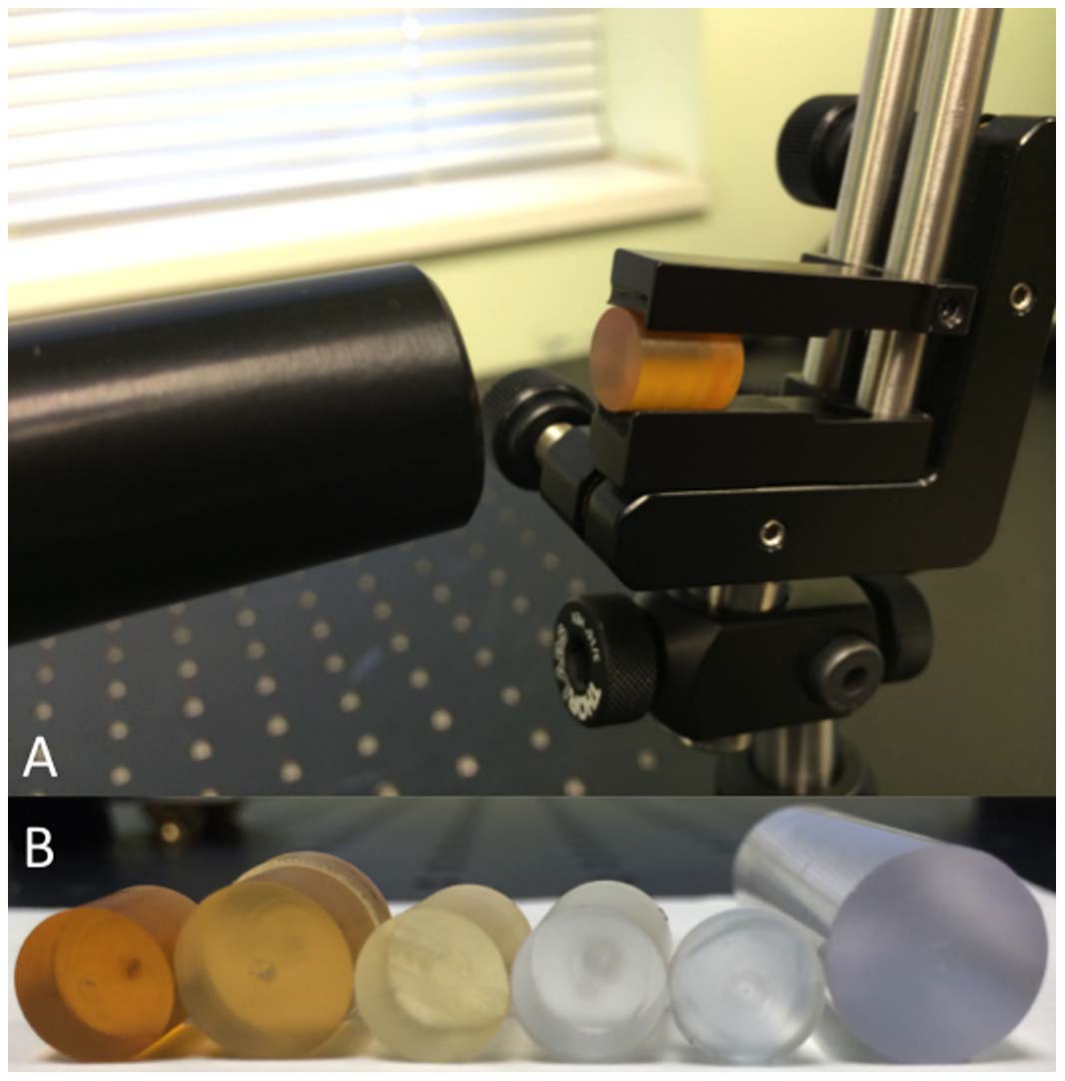
Optical testing of potential instrument materials. (A) OCT set-up with single material in place for evaluation of attenuation coefficient and scattering density. (B) Sample set of material cylinders tested for optical properties.

Six semitransparent materials were imaged using a Bioptigen SDOIS system (830±30 nm, 50 kHz line-rate). The materials were translated at 1 micron increments over a 1.5 mm range relative to a fixed focal plane, and B-scans were acquired at each position (Figure 2). Intensity changes resulting from fall-off and defocus were corrected in post-processing using experimentally measured values. Each B-scan was then averaged to remove speckle noise, and the resulting scattering profile was fit to a Beer's law model using least-squares minimization to calculate the attenuation coefficient [27]. The scattering density was calculated by measuring the percentage, by volume, of scatterers with intensities two standard deviations above the OCT noise floor.

Based on the material testing, OCT-compatible surgical implement prototypes were developed and tested. Surgical instruments tested included a corneal needle (specifically designed for deep anterior lamellar keratoplasty), surgical pick, and retinal forceps.

### Intraoperative Imaging Protocol

Various scan methods were utilized to visualize instrument motion and surgical maneuvers. As previously described, a surgical maneuver scanning protocol captured 5 B-scans, each comprised of 256 A-scans, along the axis of the instrument over a 10.0×1.0 mm FOV. The scans were oriented such that the densely sampled, long scanning axis (i.e., 10.0 mm) was aligned parallel to the axis of the surgical instrument. The direction of motion associated with the surgical maneuver was also constrained to this axis [24], [28]. These subsampled volumetric datasets were acquired at 39 volumes per second at the instrument tip and displayed in real-time for surgical guidance. The HUD system was utilized with various targets to assess localization to the area of interest. Additional scan protocols were also performed with *i*OCT B-scans oriented perpendicular to the long axis of the instrument. Post-processing included image averaging, registration, and spatial compounding with ImageJ (freeware; National Institutes of Health; Bethesda, MD) and Matlab 2010a (Matlab, MathWorks, Natick, MA).

### Model eye studies

Fresh cadaveric porcine eyes were obtained for imaging (J. H. Routh Packing, Sandusky, OH). To optimize corneal clarity and image quality, *i*OCT scanning was performed within 6 hours of harvesting the eyes. Despite efforts to acquire images immediately after enucleation, some image quality variation was observed in the surgical microscope view and on *i*OCT as a result of corneal defects and lens opacity in the cadaveric eye. Each eye was positioned under the microscope-integrated *i*OCT system using a suction platform to maintain fixation. A 23-gauge valved trocar cannula system was utilized for all posterior segment surgical maneuvers. Cannulas were placed in the standard location for 3-port vitrectomy, including infusion placement in the inferotemporal quadrant. A VersaVIT (Synergetics, St. Louis, MO) was used for infusion and vitrectomy as needed. A 23-gauge fiberoptic light pipe was used for illumination and surgical instruments were introduced through the final cannula. Initial focus was achieved by the surgeon using the BIOM or microscope pedals if a contact lens was used. Once gross focus was achieved, the tunable lens was used to for fine adjustment of the OCT image.

Volume scanning was performed prior to instrument manipulation with each instrument at multiple locations: above the cornea, within the cornea, within the anterior chamber, superficial to the retina in vitreous, and on the retinal surface. Static volumetric scans (10×10 mm) sampled at 500×500 pix. (A-scans × B-scans) were obtained for densely-sampled visualization of tissue-instrument interactions in post-processing. Dynamic imaging was performed during surgical manipulations with various instruments including a transretinal needle, diamond dusted membrane scraper (Synergetics, O'fallon, MO), vitreoretinal forceps. These maneuvers included simulated membrane peeling, vessel manipulation, cannulation of the subretinal space, subretinal intraocular foreign body removal, and corneal penetration. All simulated surgical maneuvers were performed by a trained vitreoretinal surgeon and the *i*OCT system was operated by an imaging technician adjacent to the surgical table.

## Results

### Intraoperative Imaging and Heads-up Visualization

The microscope-integrated *i*OCT system provided high-resolution images of the anterior chamber and retina in cadaveric porcine eyes. Posterior segment images were obtained with both direct and indirect lens systems, and the tunable lens provided significant flexibility and versatility for optimizing image quality. Instruments were successfully visualized in numerous anatomic locations without difficulty, including outside the eye, within the cornea, in the anterior chamber, in the vitreous cavity, and on the retinal surface.

**Figure 3:**
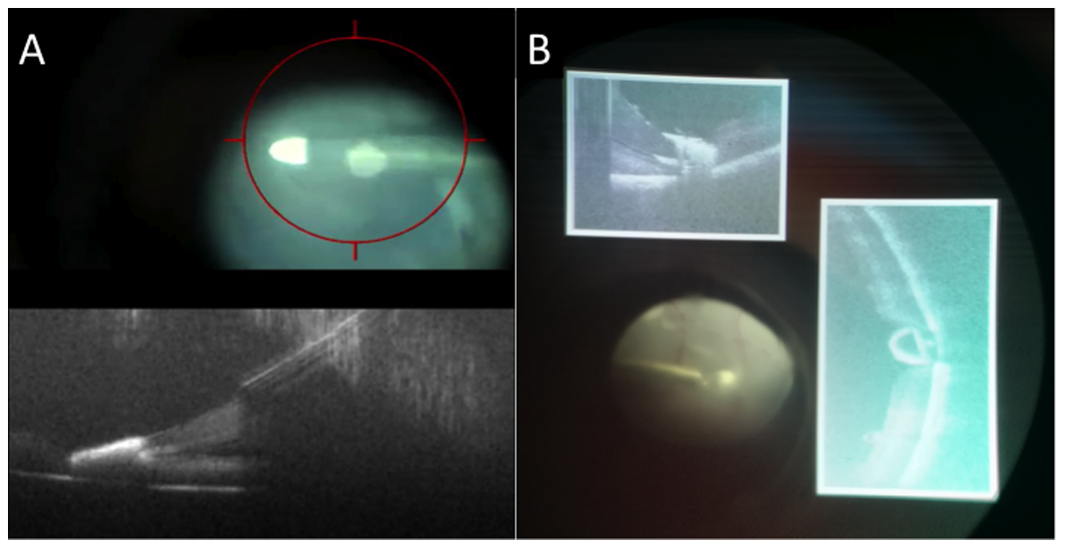
HUD system for intraoperative OCT (*i*OCT) system. (A) Ocular feedback with target region for surgeon to area of *i*OCT FOV. (B) Second ocular feedback system with OCT data displayed adjacent to surgeon's view of the surgical field with OCT display both on axis and perpendicular to instrument. Video S1. (Note: contrast non-uniformity in the *i*OCT HUD images are a result of capturing video snapshots through the surgical microscope ocular). Scale bar: 200 µm.

The HUD system was successfully provided two separate feedback mechanisms to the surgeon. The first was a targeting box to allow the surgeon to identify the coordinates of the *i*OCT scan to help co-localize the instrument-tissue interaction with the *i*OCT FOV (Figure 3A, Video S1). This provided improved efficiency and coordination between the surgeon and technician for visualizing surgical maneuvers.

The second HUD feedback mechanism provided real-time *i*OCT overlays, which allow the surgeon to simultaneously view the surgical field and *i*OCT B-scans at the area of interest (Figure 3B). This provided important feedback to the surgeon regarding depth information (e.g., corneal needle penetration) and relative location of the instrument to the tissue of interest. Software control of the HUD overlays allowed live display of *i*OCT B-scans during aiming and data acquisition, real-time scaling of *i*OCT display sizes relative to the surgical field, and review of sequential B-scans in a volumetric dataset.

### Material Testing and Surgical Instrument Prototyping

**Figure 4:**
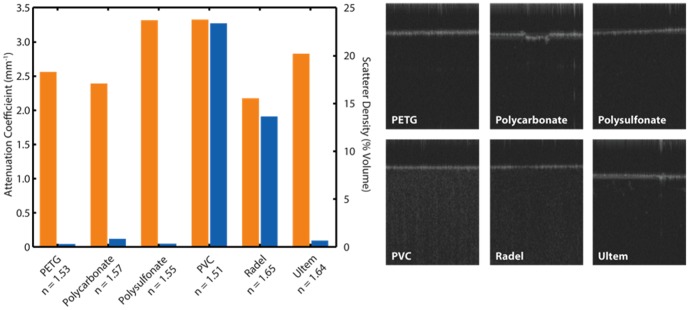
OCT-based attenuation coefficient and scattering density of potential instrument materials. The measured attenuation coefficients (yellow) ranged between 2.16–3.31 mm^−1^ and scattering densities ranged from 0.13–23.2% (blue). Index of refraction for each material at 830 nm were also measured. Representative OCT cross-sections of each material are shown.

The measured optical properties of the six semitransparent materials (polycarbonate, PVC, Radel, Ultem, polysofonate, and PETG) showed comparable attenuation coefficients with values ranging from 2.16–3.31 mm^−1^. These values were consistent with the semitransparent nature of the materials within the OCT wavelength range tested. The index of refraction for each material was also measured at 830 nm, and these values ranged between n = 1.51–1.65. The materials showed dramatically different scattering densities, ranging from 0.13–23.2% by volume, which can be clearly distinguished on OCT cross-sections (Figure 4).

**Figure 5:**
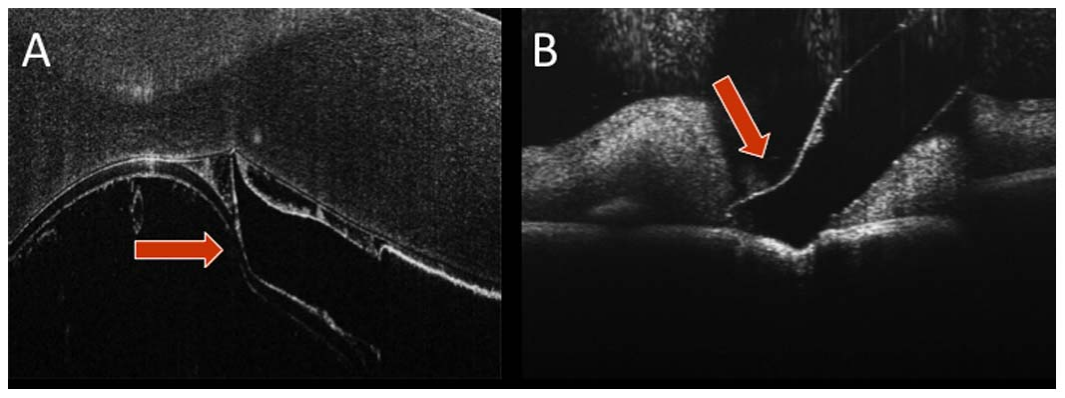
Examples of OCT-compatible instrument prototypes. (A) B-scan of prototype surgical pick (orange arrow) in the porcine anterior chamber between the endothelium and the anterior lens capsule. (B) B-scan of prototype surgical pick exerting significant downward pressure on the porcine retinal surface. In both frames, excellent visualization of the instrument profile and underlying tissues is achieved. Images show averages of 5 co-registered adjacent B-scans. Scale bar: 200 µm.

**Figure 6:**
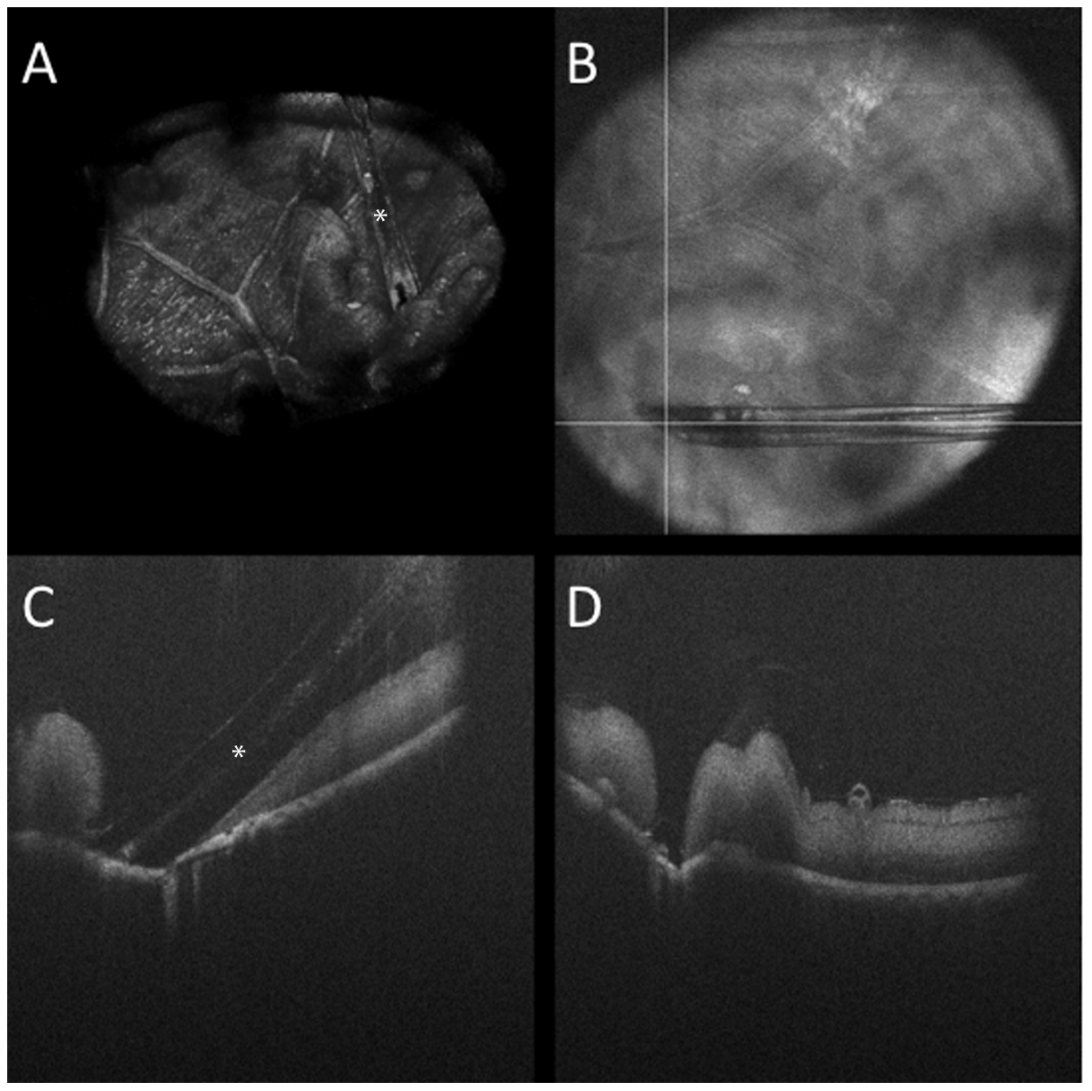
Three dimensional reconstruction of OCT-compatible needle (white asterisk) in porcine eye in (A). (B) Summed voxel projection showing perpendicular OCT scans (white lines) at needle tip. (C) Intraoperative OCT B-scan parallel to needle from (B) revealing excellent visualization of the needle while maintaining optimal clarity of the underlying tissue. The bore of the needle (white asterisk) is visible on the OCT scan. (D) Intraoperative OCT B-scan perpendicular to needle tip from (B) revealing tissue disruption at the needle tip (i.e., retinal hole) with excellent visualization of underlying and surrounding tissues. Video S2. Scale bar: 50 µ.

**Figure 7:**
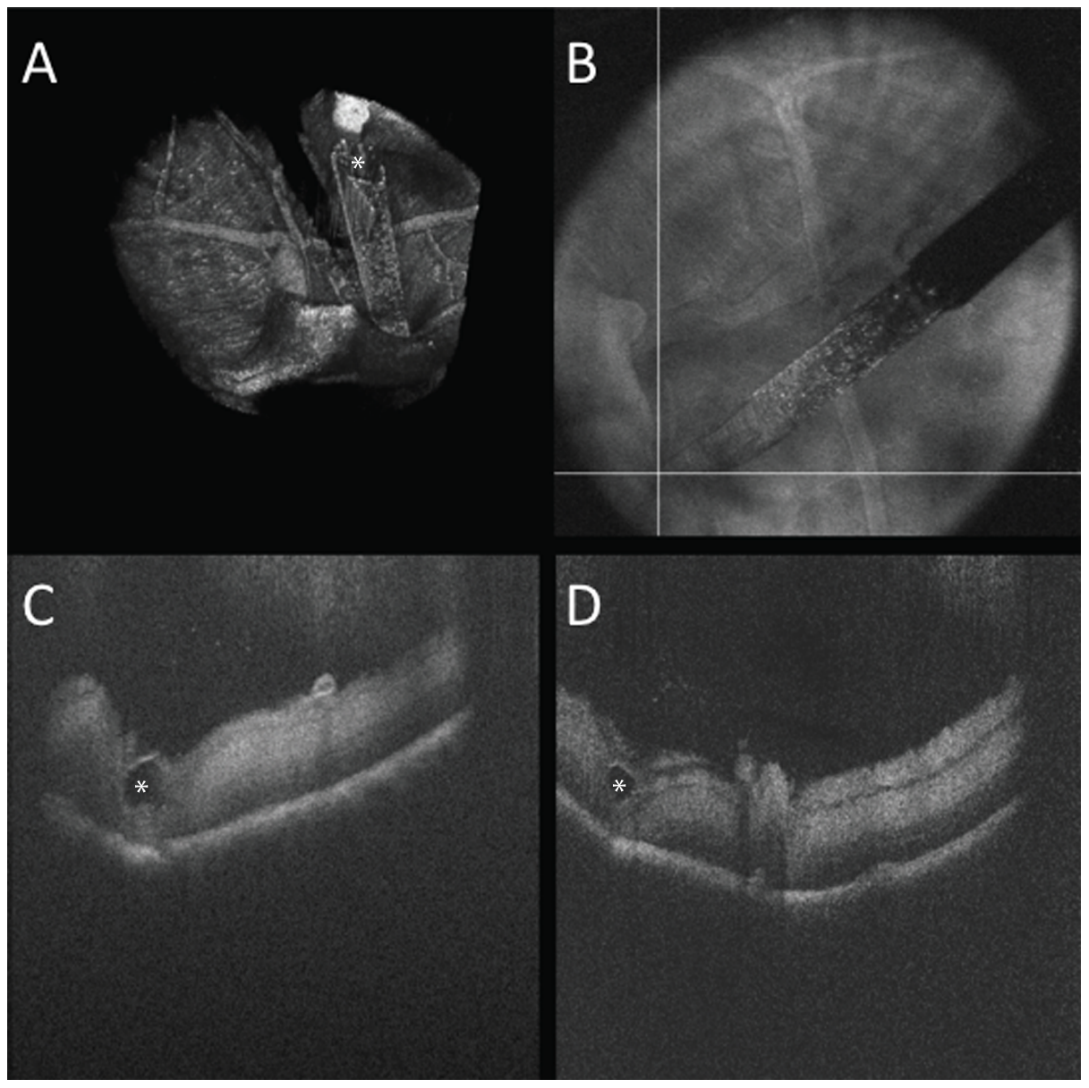
Three dimensional reconstruction of OCT-compatible surgical pick (white asterisk) in porcine eye in (A). (B) Summed voxel projection showing perpendicular OCT scans (white lines) at tip of surgical pick. (C, D) Intraoperative OCT B-scan parallel (C) and perpendicular (D) to pick from (B) revealing excellent visualization of the pick tip (white asterisk) while maintaining optimal clarity of the underlying tissue. Video S3. Scale bar: 500 µm.

Prototype instruments were successfully created, including retinal forceps, a surgical pick (Figures 5 and 6; Video S2), and an OCT-compatible needle (Figure 7; Video S3). The prototype instruments in this study were machined to accommodate a 23-gauge valved trocar cannula for simulated vitreoretinal surgery. Specifically, all instruments included a 640 µm diameter metallic shaft and 450 µm diameter semitransparent tip. The needle had an inner diameter of ∼180 µm. OCT B-scans revealed excellent visualization of the underlying tissues in addition to the tip of the instruments. This confirmed optimal light scattering and transmission properties of the substrate materials.

### Intraoperative Maneuver Visualization and Motion-Capture

**Figure 8:**
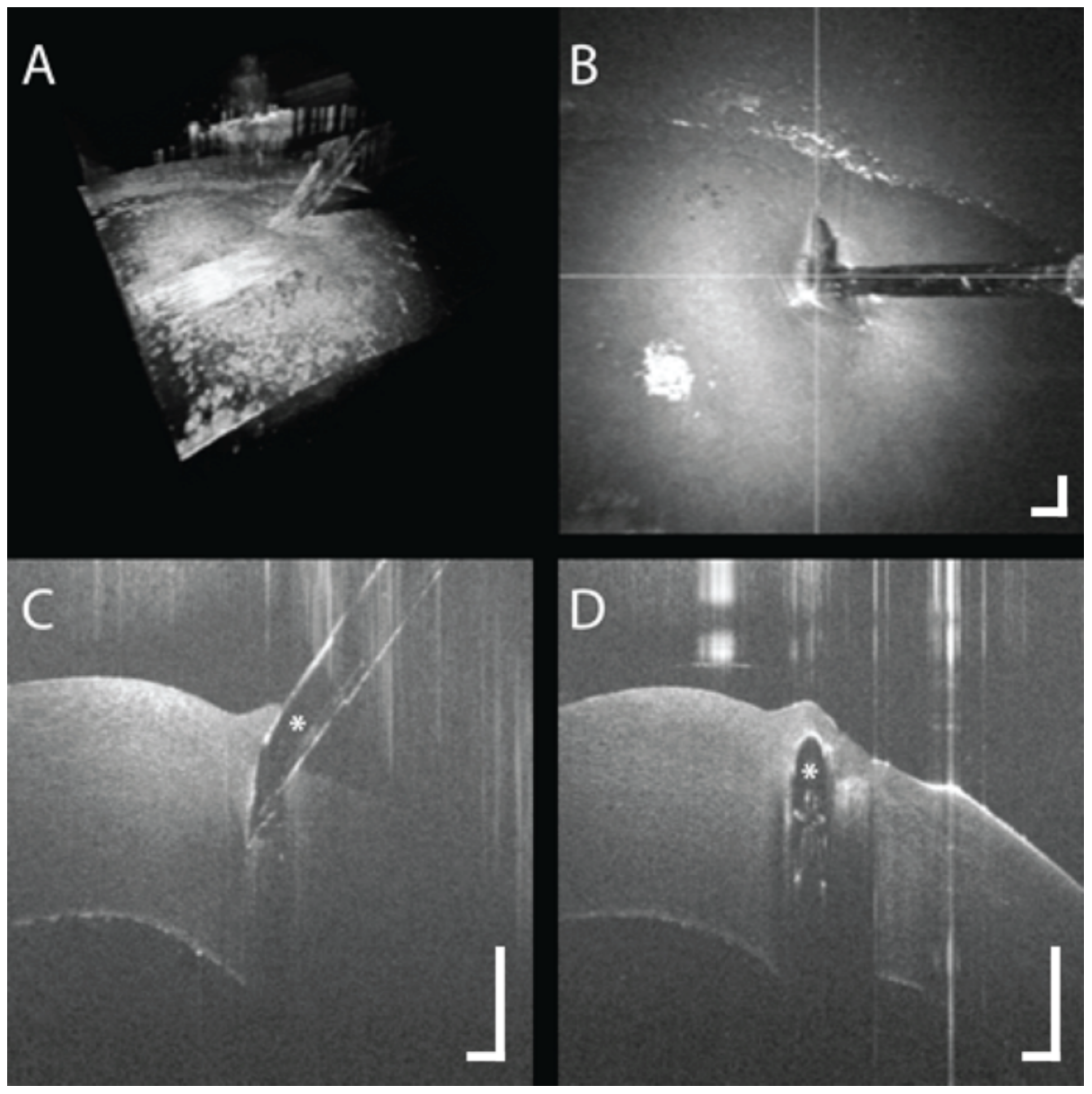
Three dimensional reconstruction of OCT-compatible needle (white asterisk) in porcine cornea in (A). (B) Summed voxel projection showing perpendicular OCT scans (white lines) at tip of needle. (C, D) Intraoperative OCT B-scan parallel (C) and perpendicular (D) to needle from (B) revealing visualization of the penetration depth of the needle tip (white asterisk) relative to the corneal endothelium. Video S4. Scale bar: 500 µm.

**Figure 9:**
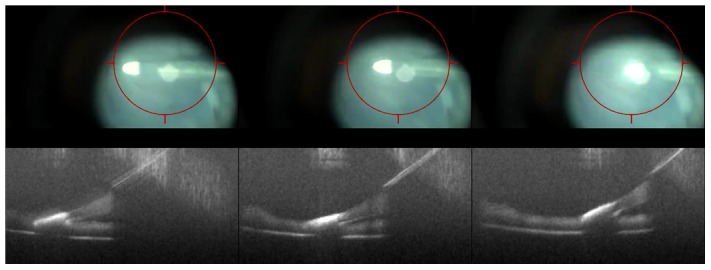
Dynamic imaging with HUD system of a diamond dusted membrane scraper on the porcine retinal surface. Each frame shows progressive OCT-motion (below) corresponding to the surgical view with HUD of OCT target (above). Video S1. Scale bar: 200 µm.

**Figure 10:**
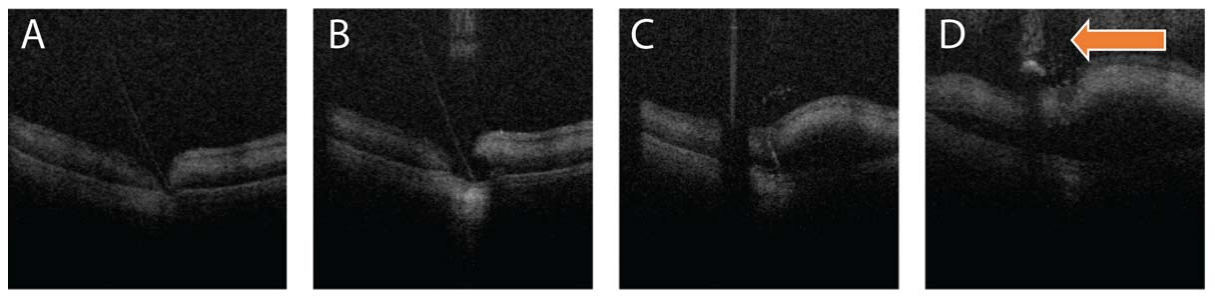
Subretinal cannulation and injection in porcine eye. Cross-sectional images show (A) semitransparent needle tip entering the subretinal space, (B) initial injection volume displacing surrounding tissue, (C) injection into the subretinal space with elevation of the retina. (D) Immediately post-injection, excess fluid and triamcinolone is observed leaking from the injection site (arrow). Video S5. Scale bar: 500 µm.

Successful imaging of conventional surgical instruments was obtained using the microscope-integrated *i*OCT system. Corneal needle penetration for simulated deep anterior lamellar keratoplasty was captured with dynamic cross-sectional and static volumetric imaging (Figure 8). Vitreoretinal procedures performed using the diamond dusted membrane scraper (Synergetics, O'fallon, MO) was imaged using spatially compounded cross-sections and densely sampled three-dimensional volumes for visualization of dynamic and static datasets in post-processing. The *i*OCT images were similar to those previously presented, showing excellent transmission through areas of bare silicone with increased shadowing at the diamond dusted tip [24], [25]. Utilizing the HUD system, images of the membrane scraper could be rapidly obtained, and dynamic imaging was used to demonstrate intrasurgical instrument motion on the retinal surface (Figure 9, Video S1). Additional simulated vitreoretinal surgical maneuvers captured with dynamic imaging included membrane peeling, vascular manipulation, subretinal intraocular foreign body removal, and subretinal space cannulation with injection of triamcinolone (Figure 10, Video S5). Spatial compounding enhanced surgical instrument visualization and minimized underlying shadowing.

## Discussion

In this study, we demonstrated several iterative advances in integrative technology for *i*OCT. Application of a HUD system represents a major advance in integrative technology. The ability to visualize *i*OCT data without the use of an external monitor significantly enhanced ergonomics by providing immediate feedback to surgeons through the microscope oculars without needing to look away from the surgical field. In addition to the *i*OCT system, we characterized the optical properties of several materials and demonstrated the feasibility of OCT-compatible instrumentation, which improves visualization of underlying tissue structures and instrument-tissue interactions.

Dynamic imaging with spatial compounding provided outstanding visualization of numerous ophthalmic surgical maneuvers. However, spatial averaging over adjacent B-scans, acquired across the width of a surgical instrument, limits high-speed visualization of surgical dynamics and may introduce averaging artifacts. Recent development of novel high-speed OCT light sources [29], [30] and real-time volumetric OCT processing and visualization algorithms [31], [32] may potentially allow for real-time acquisition and visualization of volumetric datasets for imaging intraoperative maneuvers. However, visualization of three-dimensional data conventionally requires real-time rotation of rendered datasets and serial fly-throughs of cross-sectional images, which may prove cumbersome and distracting for surgical guidance. Potential visualization methods may include automated surgical instrument tracking for *i*OCT or HUD overlays of *en face* instrument positions calculated using segmentation of retinal layers and instrument surfaces. Future studies are required to explore the utility of different methods for intraoperative guidance.

Our preliminary testing indicates that materials with minimal attenuation and refractive index are optimal candidates for OCT-compatible instruments. Minimal attenuation results in reduced signal loss and shadowing of tissue structures underneath the instrument, and low refractive index results in smaller axial image jump as a result of optical pathlength differences between the instrument material and air/vitreous. Initial prototype instruments were machined from polycarbonate, but PETG may also be an appropriate candidate material because of similarities in their optical properties at the OCT wavelength range tested. Scatterer density may also be taken into account because a combination of low attenuation and high scattering may provide enhanced OCT contrast. However, machining methods, such as surface roughing, or adding scattering dopants into the substrate material may allow for more flexibility in designing specific scattering properties into OCT-compatible instruments.

Vitreoretinal surgery has made remarkable progress over the last few decades. Advances in surgical microscopes, small-gauge instrumentation, illumination and vital dyes have resulted in significant improvements in the ophthalmic surgeon's toolbox and patient outcomes [33]–[36]. As OCT has transformed the landscape of clinical ophthalmology, true seamless integration of *i*OCT into the operating room may be the next paradigm shift in ophthalmic surgery.

Preliminary research suggests that *i*OCT may provide critical information regarding the impact of surgical maneuvers, intrasurgical architectural dynamics, and pathophysiology of surgical diseases and may impact surgical decision-making [2]–[6], [8], [37]. Using *i*OCT, studies have examined multiple vitreoretinal diseases including vitreomacular traction syndrome, macular holes, retinal detachment, epiretinal membrane, optic pit-related maculopathy, and ROP [2], [3], [5], [8], [23], [37], [38]. The dynamic nature of surgery provides a unique opportunity to study the pathophysiology of underlying diseases and architectural changes that occur during surgical manipulation. These underlying dynamics have been demonstrated with *i*OCT for optic pit-related maculopathy, retinal detachment, and macular holes [2], [4], [37].

Utilizing information obtained from *i*OCT, surgeon decision-making may be altered and patient outcomes may be impacted. The impact of *i*OCT on surgeon decision-making has been demonstrated in vitreomacular traction and epiretinal membrane surgery (Ehlers et al, IOVS 2013. 54: ARVO E-abstract 3308) [3]. In these reports, *i*OCT demonstrated potential impact on patient outcomes by aiding surgical decision-making (e.g., additional membrane peeling, gas tamponade). Additionally, feedback during deep anterior lamellar keratoplasty regarding dissection depth may be helpful in optimizing the procedure [13].

In order for *i*OCT to become a key component of ophthalmic surgery, significant improvements are needed in integration to minimize disruption to work-flow while maximizing surgical feedback. Previously described microscope-integrated OCT systems were landmark advances for integrative solutions [5], [20]. However, significant shortcomings still existed in the system-surgeon interaction platform. Namely, HUD systems, OCT-compatible instrumentation, automated aiming, customizable focus control, and software analysis platforms were all lacking.

In this report, we demonstrate significant iterative progress for many of these hurdles, particularly related to focus control, HUD systems, and OCT-compatible instrumentation. We have previously demonstrated novel algorithms for pathology segmentation that may be applied to identify the rapid alterations that occur during surgical intervention [4], [39]. Automated segmentation of retinal layers has been previously described [40]. The application of similar algorithms could potentially be utilized for real-time surgical feedback on instrument-tissue proximity. Automated layer segmentation could potentially be utilized to track intra-tissue depth and open doors to new surgical procedures (e.g., targeted drug delivery).

The systems described in this report represent significant but preliminary advances in integrative technology. Additional progress is needed in automated aiming and tracking of surgical instrumentation. Tailored image-feedback is also needed to optimize the HUD system for optimal performance and minimizing information overload. The OCT-compatible instruments represent proofs-of-concept and still need additional iterations to maximize functionality and expand the surgical tool repertoire. Specifically, material properties will need to be rigorously examined to identify differences between the semi-transparent plastics identified in this manuscript versus conventional metallic instruments. These material properties, including hardness and tensile strength, will be critical in identifying substrates that may be easily manufactured and machined to tolerances acceptable for surgical implementation, specifically in instrument precision and durability. Our simulated corneal surgery using an OCT-compatible needle identified potential differences in the overall feel compared to conventional metallic needles, which will need to be addressed in future studies.

From femtosecond cataract surgery to ultra-high speed vitrectomy, intrasurgical technology has experienced significant growth in the past several years. *i*OCT is an emerging technology, and interest in the application of OCT to surgical care continues to gain momentum. Currently, two microscope-integrated systems have approval in the EU for human use: the Haag-Streit *i*OCT and Carl Zeiss Meditec RESCAN 700. The Haag-Streit *i*OCT system utilizes a camera port add-on OCT system from OPMedT [9]. Carl Zeiss Meditec recently developed a second-generation *i*OCT prototype, the RESCAN 700, that is built on the Lumera 700 platform [9], [23]. The Haag-Streit system uses an external monitor, whereas the RESCAN 700 provides a HUD system for surgical use. At the time of this report, neither of these systems is FDA-approved in the United States.

*i*OCT represents an exciting opportunity for expansion of our knowledge base of surgical ophthalmic conditions and a potential paradigm shift in ophthalmic surgical management. This study demonstrates subsequent integrative advances that may be critical to the widespread assimilation of this technology into every day surgical practice, including an optimized form-factor, OCT-compatible instrumentation, and a HUD display feedback. Further research and development is needed to develop software platforms, automated instrument tracking, and surgical instruments in order to continue to optimally integrate this technology seamlessly into the operating room theater.

## Supporting Information

Video S1 **Heads-up display feedback for intraoperative OCT (** ***i*** **OCT) system.** Microscope ocular feedback with target region for surgeon to area of *i*OCT field of view (top). Simultaneous *i*OCT video of instrument-tissue interaction (bottom).
(MOV)

Video S2 **Three dimensional reconstruction of OCT-compatible needle in porcine eye (top left).** Summed voxel projection showing perpendicular OCT scans at needle tip (top right). Intraoperative OCT B-scan, parallel to needle, reveals excellent visualization of the needle while maintaining optimal clarity of the underlying tissue (bottom left). Intraoperative OCT B-scan, perpendicular to needle tip, reveals tissue disruption at the needle tip (i.e., retinal hole) with excellent visualization of underlying and surrounding tissues (bottom right).
(MOV)

Video S3 **Three dimensional reconstruction of OCT-compatible surgical pick in porcine eye (top left).** Summed voxel projection showing perpendicular OCT scans at tip of surgical pick (top right). Intraoperative OCT B-scan parallel (bottom left) and perpendicular (bottom right) to surgical pick revealing excellent visualization of the pick tip while maintaining optimal clarity of the underlying tissue.
(MOV)

Video S4 **Three dimensional reconstruction of OCT-compatible needle in porcine cornea (top left).** Summed voxel projection showing perpendicular OCT scans at tip of needle (top right). Intraoperative OCT B-scan parallel (bottom left) and perpendicular (bottom right) to needle captures the penetration depth of the needle tip relative to the corneal endothelium.
(MOV)

Video S5 **Subretinal cannulation and injection in porcine eye.** Cross-sectional images show semitransparent needle tip entering the subretinal space, initial injection volume displacing surrounding tissue, injection into the subretinal space with elevation of the retina. Immediately post-injection, excess fluid and triamcinolone is observed leaking from the injection site.
(MOV)
